# An autoinflammatory syndrome with compound heterozygous *MEFV* and *NOD2*/*CARD15* gene mutations successfully treated with tocilizumab

**DOI:** 10.1093/rap/rkac035

**Published:** 2022-05-09

**Authors:** Jeanie Lee, Lilian Bizzocchi, Ruchi Jain, Clement E Tagoe

**Affiliations:** 1 Department of Medicine, Albert Einstein College of Medicine, Montefiore Medical Center, Bronx, NY; 2 Department of Rheumatology, CoxHealth, Springfield, MO, USA

Key message

- Novel heterozygous *MEFV* and *NOD2* gene mutations cause an autoinflammatory syndrome with colchicine resistance and tocilizumab sensitivity.

Dear Editor, Autoinflammatory syndromes are characterized by systemic inflammation in the absence of infections and include FMF, an inherited disorder characterized by recurrent fevers and serositis [1]. It presents during the first or second decade of life but can present later in 0.5% of cases [2]. Inheritance is autosomal recessive, with the causative mutation in the *MEFV* gene. Heterozygous transmission can constitute a susceptibility factor for clinically similar multifactorial forms of the disease [3]. The *NOD2*/*CARD15* gene is associated with chronic inflammatory conditions, such as Crohn’s disease, Blau syndrome, Yao syndrome and early-onset sarcoidosis [4]. Recently, the *NOD2*/*CARD15* gene has been suggested as a disease modifier for *MEFV* autoinflammatory disease, causing a more severe form of FMF-like phenotype with a higher rate of colchicine resistance [4, 5]. We describe a case of an autoinflammatory syndrome with a FMF-like presentation characterized by recurrent fevers, colchicine resistance, tocilizumab sensitivity and novel compound heterozygous mutations in the *MEFV* and *NOD2* genes.

A 44-year-old Hispanic woman of Albanian descent weighing 63 kg presented with recurrent fevers, diffuse lymphadenopathy and abdominal pain for 1 year. Each febrile episode was characterized by fevers to 38.9°C that lasted 1–9 days, followed by intervals of 4–5 days without fevers. She had no family history of autoinflammatory diseases. Previously, she had presented with similar symptoms. CT of the abdomen and pelvis showed retroperitoneal and mesenteric lymphadenopathy. She received a course of antibiotics. One month later, she returned with similar symptoms. CT-guided retroperitoneal lymph node biopsy and diagnostic laparoscopic mesenteric lymph node biopsy showed reactive lymphoid hyperplasia. Bone marrow biopsy was normocellular. At this admission, she was febrile to 39.4°C. Abdominal examination demonstrated diffuse guarding without rebound tenderness. Laboratory testing revealed haemoglobin of 10.4 g/dl, elevated ESR [93 mm/h (normal < 20mm/h)], elevated CRP [3.8 mg/dl (normal <0.8 mg/dl)] and elevated serum angiotensin converting enzyme [>80 U/l (normal < 50U/l)]. Her CT was unchanged (Fig. 1).

**Fig. 1:**
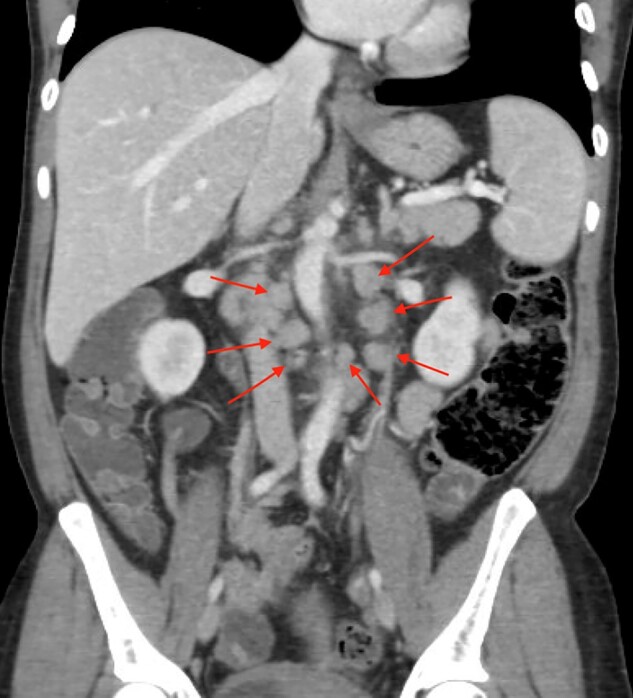
CT of the abdomen and pelvis showing lymphadenopathy (indicated by red arrows) in the retroperitoneum, with left para-aortic nodes as large as 2 cm and with multiple nodes at the base of the small bowel mesentery and multiple right and left para-aortic nodes

Her differential diagnosis included malignancy, infection and autoimmune disease. Retroperitoneal lymph nodes raised the possibility of IgG4-related disease, but IgG4 subclasses were normal, and biopsies lacked the characteristic histological features. Adult-onset Still’s disease appeared less likely given normal ferritin and lack of its distinct rash with her fevers. Elevated inflammatory markers and angiotensin converting enzyme were suggestive of sarcoidosis, but the patient did not have hilar lymphadenopathy, erythema nodosum or non-caseating granulomas. Cleavage-resistant RIPK1-induced autoinflammatory (CRIA) syndrome typically presents with periodic fevers, lymphadenopathy and anaemia. However, symptoms start from childhood. Genetic study of monogenic autoinflammatory diseases was ordered.

Multidisciplinary discussions deemed repeat lymph node biopsy to be invasive owing to the proximity of nodes to vital structures. A pathological review of previous histological data favoured follicular hyperplasia and reactive histiocytosis of the lymph nodes. IgG4 staining was negative. The patient was discharged with close outpatient follow-up.

She was readmitted with fever and abdominal pain 2 months later. The genetic study for monogenic autoinflammatory diseases that examined 10 genes revealed two heterozygous variants of uncertain significance, namely c.1522C>G (p.Leu508Val) in the *MEFV* gene and c.274G>T(p.Val92Phe) in the *NOD2* gene. Neither has been reported pathogenic in Infevers, a registry for autoinflammatory mutations at https://infevers.umai-montpellier.fr/web/ [1], or other genomic databases. A presumed diagnosis of FMF-like autoinflammatory syndrome was made, and an empirical trial of colchicine was initiated at 0.6 mg daily, then escalated to 0.6 mg three times daily, but with modest response. Her cytokine panel showed elevated IL-6 of 20.66 pg/ml (normal < 5 pg/ml) and low IL-1β of <5 pg/ml (normal ≤ 36 pg/ml). An IL-6 inhibitor, tocilizumab, was added for possible colchicine resistance. Her fevers resolved within 1 month, and she has remained asymptomatic for 1 year, with improvement in inflammatory markers.

The coexistence of *MEFV* and *NOD2* gene variants has been reported in the literature more often than other genetic combinations [4]. FMF patients with *NOD2*/*CARD15* gene mutations can present with a more severe form, with a higher rate of colchicine resistance [5], suggesting that the coexistence of these digenic variants can cause a phenotypic modification [4]. This hypothesis might explain the atypical presentation of FMF-like disease in our patient, including older age of onset, clinical expression of heterozygous state, atypical fever patterns and retroperitoneal lymphadenopathy, reported in only five other cases [6]. The *NOD2* gene variant might also explain her intolerance to colchicine that warranted escalation of therapy. This case identifies novel heterozygous variants in the *MEFV* and *NOD2* genes that might have pathogenic significance in autoinflammatory syndromes. It also underscores the importance of considering autoinflammatory diseases in unexplained chronic fever, because compound genetic variations can lead to modifications in clinical phenotype [7]. Future work should catalogue similar cases of compound heterozygous mutations of *MEFV* and *NOD2*/*CARD15* to define the influence of genetic variation on disease presentation of autoinflammatory syndromes.

Tocilizumab is an anti-IL-6 receptor antagonist that has been shown to be effective in treating colchicine-resistant FMF and secondary amyloidosis [8]. This is the first case, to our knowledge, that showed successful treatment with tocilizumab in a patient with coexisting variants of *MEFV* and *NOD2* genes. IL-1 blockade is the preferred biologic therapy for inflammasome-mediated autoinflammatory diseases and remains a therapeutic option for this patient [8].

*Funding:* No specific funding was received from any bodies in the public, commercial or not-for-profit sectors to carry out the work described in this article.

*Disclosure statement:* The authors have declared no conflicts of interest.

*Consent:* The patient provided informed consent for the publication of this manuscript.

## Data availability statement

Data are available upon reasonable request by any qualified researchers who engage in rigorous, independent scientific research, and will be provided following review and approval of a research proposal and Statistical Analysis Plan (SAP) and execution of a Data Sharing Agreement (DSA). All data relevant to the study are included in the article.
